# Mucinous tubular and spindle cell carcinoma and solid variant papillary renal cell carcinoma: a clinicopathologic comparative analysis of four cases with similar molecular genetics datum

**DOI:** 10.1186/s13000-014-0194-8

**Published:** 2014-12-05

**Authors:** Yanling Zhang, Xiang Yong, Qiong Wu, Xiaoli Wang, Qiong Zhang, Shiwu Wu, Donghong Yu

**Affiliations:** Department of Pathology, the Frist Affiliated Hospital of Bengbu Medical College, 287 Changhuai Road, 233000 Bengbu, Anhui PR China

**Keywords:** Carcinoma, Renal cell, Mucinous tubular and spindle cell carcinoma, Fluorescence in situ hybridization, Immunohistochemistry

## Abstract

Mucinous tubular and spindle cell carcinoma (MTSC) was first recognized as a specific entity in the World Health Organization 2004 classification. The “classic” tumor presentation includes an extracellular blue-gray mucinous/myxoid matrix accompanying the typical tubular and spindle cell epithelial components. Tubules are lined by cuboidal to columnar cells with bland nuclei, central small to medium sized nucleoli, and few to no mitoses. By expanding the histologic spectrum, a number of studies highlighted the distinction between MTSC and solid variant of papillary renal cell carcinoma (sPRCC), although controversy still exists. Here, we evaluated two cases of MTSC and compared two cases of sPRCC by light microscopy, special staining, immunohistochemical staining and fluorescence in situ hybridization (FISH). We found that morphologic and immunophenotyping features showed more overlap between MTSC and sPRCC. In addition, gains of chromosomes 7 and 17 and loss of Y, which are characteristic of PRCC, were observed in two cases of sPRCC and one case of MTSC, suggesting that MTSC is similar to sPRCC or may be a subtype of PRCC.

**Virtual Slides:** The virtual slide(s) for this article can be found here: http://www.diagnosticpathology.diagnomx.eu/vs/13000_2014_194

## Background

Mucinous tubular and spindle cell carcinoma (MTSC) was first recognized as a specific entity in the World Health Organization 2004 classification [1]. The “classic” tumor presentation includes an extracellular blue-gray mucinous/myxoid matrix accompanying the typical tubular and spindle cell epithelial components. Tubules are lined by cuboidal to columnar cells with bland nuclei, central small to medium sized nucleoli, and few to no mitoses.

Solid variant of papillary renal cell carcinoma (sPRCC) is a rare variant of papillary renal cell carcinoma that was first recognized by Renshaw *et al.* [2]. Whole tumor cells are arranged in solid sheets or tubular structures and foci are tightly packed mimicking spindle cells, with no true papillary structure or <20% of the volume of the tumor [3,4].

With the expansion of the histologic spectrum, MTSC with unusual morphology has also been reported, such as “mucin-poor” MTSC [5], “MTSC with prominent papillary component” [6], “spindle cell predominant” or “tubular predominant” MTSC [7], psammoma bodies and macrophages are detected in the stroma [5,7]. Morphologic overlap between MTSC and sPRCC has been reported, and a number of studies highlighted the distinction between MTSC and sPRCC, such as Paner *et al.* [8], who considered that MTSC and sPRCC with sarcomatous change can be distinguished by the spindle cell component with atypia. Argani *et al*. [9] reported five cases of sPRCC accompanying low-grade spindle cell tumors, a morphology that is difficult to distinguish from MTSC, however molecular genetic studies revealed gains of chromosomes 7 and 17 and loss of the Y chromosome, supporting the diagnosis of sPRCC. Fine *et al*. [10] suggested that MTSC and sPRCC have a morphologic and immunophenotypic overlap, but molecular genetics studies could distinguish between MTSC and sPRCC. Shen *et al.* [7] suggested that MTSC is a subtype of PRCC based on morphological and immunohistochemical results. However, there is still ambiguity in the relationship between MTSC and sPRCC [11]. Therefore, we compared two cases of MTSC and two cases of sPRCC in terms of morphology, immunohistochemistry and molecular genetics, and explored the relationship between MTSC and sPRCC.

## Case presentation

**Figure 1:**
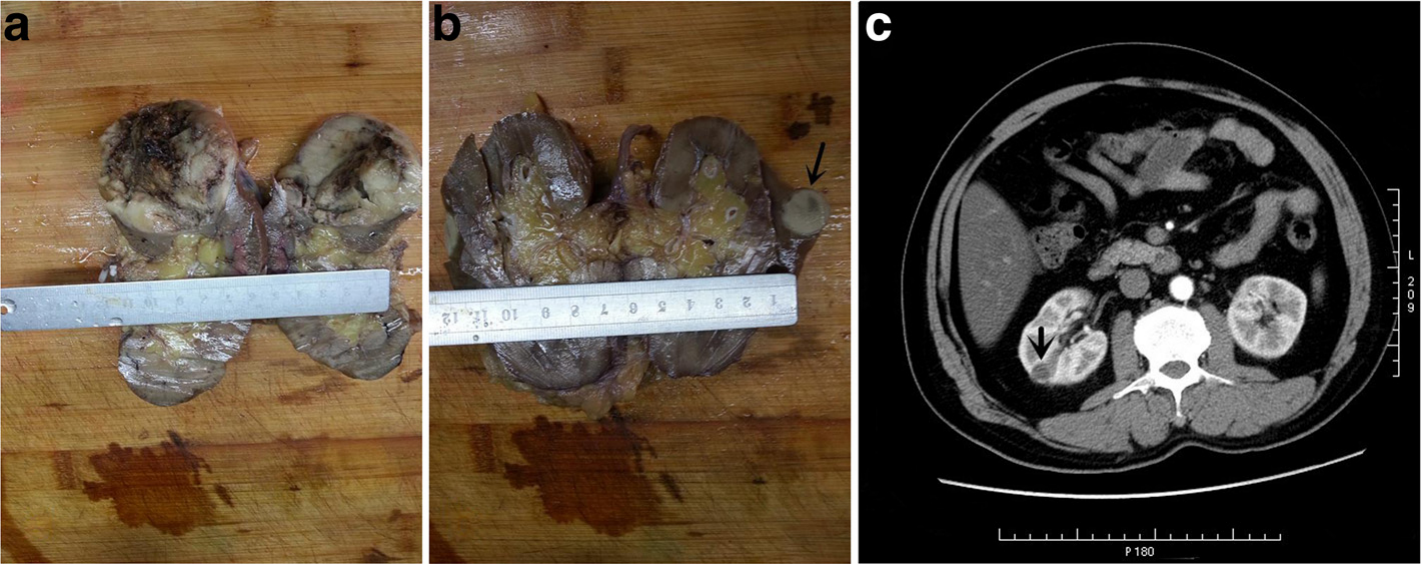
**Macroscopic observation and CT findings of MTSC and sPRCC. (a)**: MTSC (case 2) showed a well-defined nodular mass with a greyish-white cut surface accompanied with hemorrhage and necrosis. **(b)**: sPRCC (case 3) showed a well-defined nodular mass with a greyish-white cut surface (arrow). **c)**: Computed tomography revealed a mass in the renal cortex in case 3 that was well-defined (arrow).

Table 1 lists the demographic and clinical information for the MTSC and sPRCC cases examined. Case 1 (MTSC) was a 71 year-old man admitted to the hospital for flank pain. Ultrasound showed a substantive mass suspicious of renal carcinoma. Another MTSC patient (case 2) was a 75 year-old woman, who presented with computed tomography findings showing a solid mass in the right kidney that was considered potentially malignant. Two cases of sPRCC (cases 3 and 4) were men, 50 and 51 years-old, respectively. Computed tomography showed that case 3 had a space-occupying lesion in the right kidney that was considered potentially malignant and case 4 had a substantive lesion in the right kidney. Radical resection of the kidney was performed in all four cases. All four cases showed well-defined nodular masses of 3.5, 6.0, 2.5 and 8.0 cm in diameter. The cut surfaces were greyish-white in color. Cases 1 and 3 showed hemorrhage and necrosis in central regions. Cases 1, 2, and 3 were staged as T1 tumors and case 4 as T2 (Figure 1, Table 1).

**Table 1:** **Clinicopathologic features of 4 cases**

**Tumor type**	**Case**	**Age**	**Sex**	**Tumor size(cm)**	**Stage**	**Follow-up(mo)**
MTSC	1	71	Male	3.5 cm	T1aN0	14
	2	75	Female	6.0 cm	T1bN0	13
sPRCC	3	50	Male	2.0 cm	T1aN0	12
	4	51	Male	8.0 cm	T2N0	8

## Methods

### Immunohistochemistry and special staining

The collected specimens were fixed with 10% neutral buffered formalin and embedded in paraffin blocks. Tissue blocks were cut into 4-μm slides, deparaffinized in xylene, rehydrated with a graded alcohol series, and immunostained with the following antibodies: AMACR/P504S, CK, CK7, CK19, vimentin, CK (H), EMA, E-cadherin, CD10, CD15, RCC, CD56, NSE, Syn, CgA and villin. Sections were stained using a streptavidin-peroxidase system (KIT-9720, Ultrasensitive TM S-P, MaiXin, China). The chromogen used was diaminobenzidine tetrahydrochloride substrate (DAB kit, MaiXin), and sections were slightly counterstained with hematoxylin, dehydrated and mounted. Histochemical staining for Alcian blue at pH 2.5 was performed on sections from a block representing the predominant histology of each case. Immunohistochemical data are summarized in Table 2.

**Table 2:** **Sources and dilutions of the antibodies used in immunohistochemistry**

RCC	Monoclonal, clone PN-15,1:20(MaiXin, China)
AMACR	Monoclonal, clone 13H4,1:100(MaiXin, China)
CK	Monoclonal, clone AE1/AE3,1:100(MaiXin, China)
CK7	Monoclonal, clone OV-TL12/30,1:40(MaiXin, China)
CK19	Monoclonal, clone A53-B/A2.26,1:25(MaiXin, China)
vimentin	Monoclonal, clone V9,1:20(MaiXin, China)
HMWK	Monoclonal, clone 34βE12,1:10(MaiXin, China)
EMA	Monoclonal, clone E29,1:200(MaiXin, China)
E-cadherin	Monoclonal, clone 4A2C7,1:100(MaiXin, China)
CD10	Monoclonal, clone 56C6,1:10(MaiXin, China)
Ki-67	Monoclonal, clone MIB-1,1:100(MaiXin, China)
CD15	Monoclonal, clone Carb-3,1:100(MaiXin, China)
villin	Monoclonal, clone CWWB1,1:25(MaiXin, China)
CD56	Monoclonal, clone 56C04,1:20(MaiXin, China)
NSE	Monoclonal, clone E27, 1:100(MaiXin, China)
CgA	Monoclonal, clone 5p12, 1:100(MaiXin, China)
Syn	Monoclonal, clone SYP02 ,1:40(MaiXin, China)

### Fluorescence in situ hybridization and in situ hybridization analysis

The four cases were examined for their cytogenetic profile. A combination of probe 1 [CSP3 (green), CSP7 (blue) and CSP17 (red)] and centromeric probe 2 for chromosomes X (green) and Y (red) (Guangzhou LBP Medical Science Technology Co., Ltd., Guangzhou, China) was used. Each probe was diluted at 1:100 in tDenHyb1 buffer (Guangzhou LBP Medical Science Technology Co., Ltd.). Ten microliters of diluted probe was applied to each slide, and coverslips were placed over the slides. Denaturation was achieved by incubating the slides at 80°C for 10 min in a humidified box, and hybridization was performed at 37°C for 3 h. The coverslips were then removed, and the slides were immersed at room temperature in 0.5× SSC for 2 min, in 50% formamide/1× SSC for 5 min, and in 2× SSC for 2 min. The slides were air-dried and counterstained with 10 μL DAPI/Antifade (DAPI in Fluorguard, 0.5 μg/mL; Insitus, Albuquerque, NM, USA). The slides were examined using an Olympus IX-50 microscope (Olympus, Tokyo, Japan) with filters. Probe 1 was used for detection of chromosomes 7 (blue) and 17 (red), and CSP3 (green) was used as internal control. Probe 2 was used for detection of chromosome Y (red), and the centromeric probe X (green) was used as internal control.

Only individual and well-delineated cells were scored. Overlapping cells were excluded from the analysis. Approximately 60 tumor cells were analyzed in the targeted region. Using established criteria [12], chromosomal gains were considered significant if present in >20% of cells. Gains were considered artifactual if seen in <20% of cells. We used normal kidney tissue to determine the cutoffs. None of the control kidneys in our study demonstrated trisomic cells.

This study was approved by the Ethics Committees of the First Affiliated Hospital of Bengbu Medical College and conducted in accordance with the ethical guidelines of the Declaration of Helsinki.

## Results

**Figure 2:**
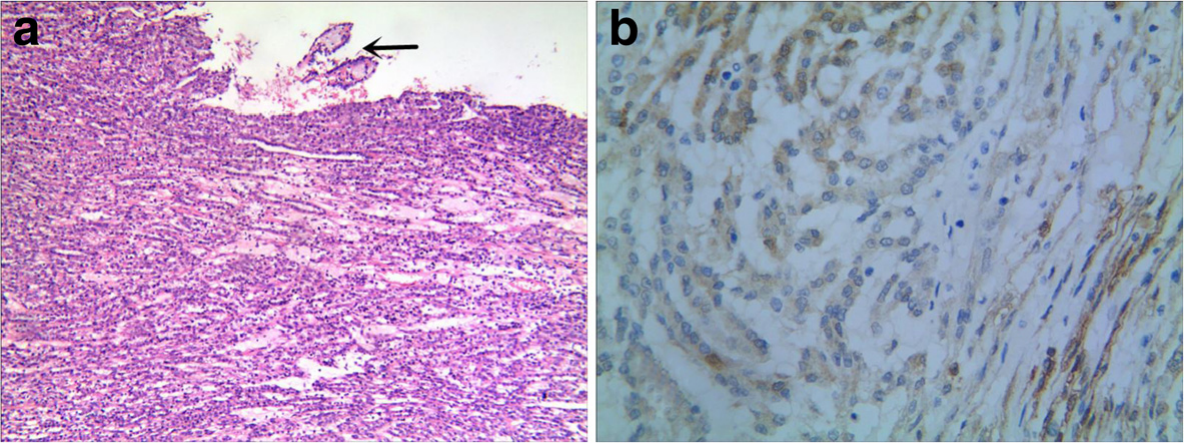
**Histological and immunohistochemical findings of MTSC. (a)**: the tumor cells are arranged in tubules accompanying spindle cell epithelial components and extracellular mucinous/myxoid matrix were significant; foci papillary structure was seen in case 1 (arrow). **(b)**: Tumor cells were positive for NSE in cases of MTSC.

Histologically, two cases of MTSC showed “classical” morphology. Both of them were predominantly composed of tubular areas with a focal spindle cell appearance, tubules were lined by cuboidal cells with bland nuclei, central small-to-medium sized nucleoli, Fuhrman nuclear grade 2 and few to no mitoses. Extracellular mucinous/myxoid matrix were prominent. Foci papillary structure was seen in case 1 with accumulation of foam cells and the accumulation of foam cells could also be seen in the mucinous stroma; lymphoid aggregates were observed in case 2. No cases had psammoma bodies (Figure 2).

**Figure 3:**
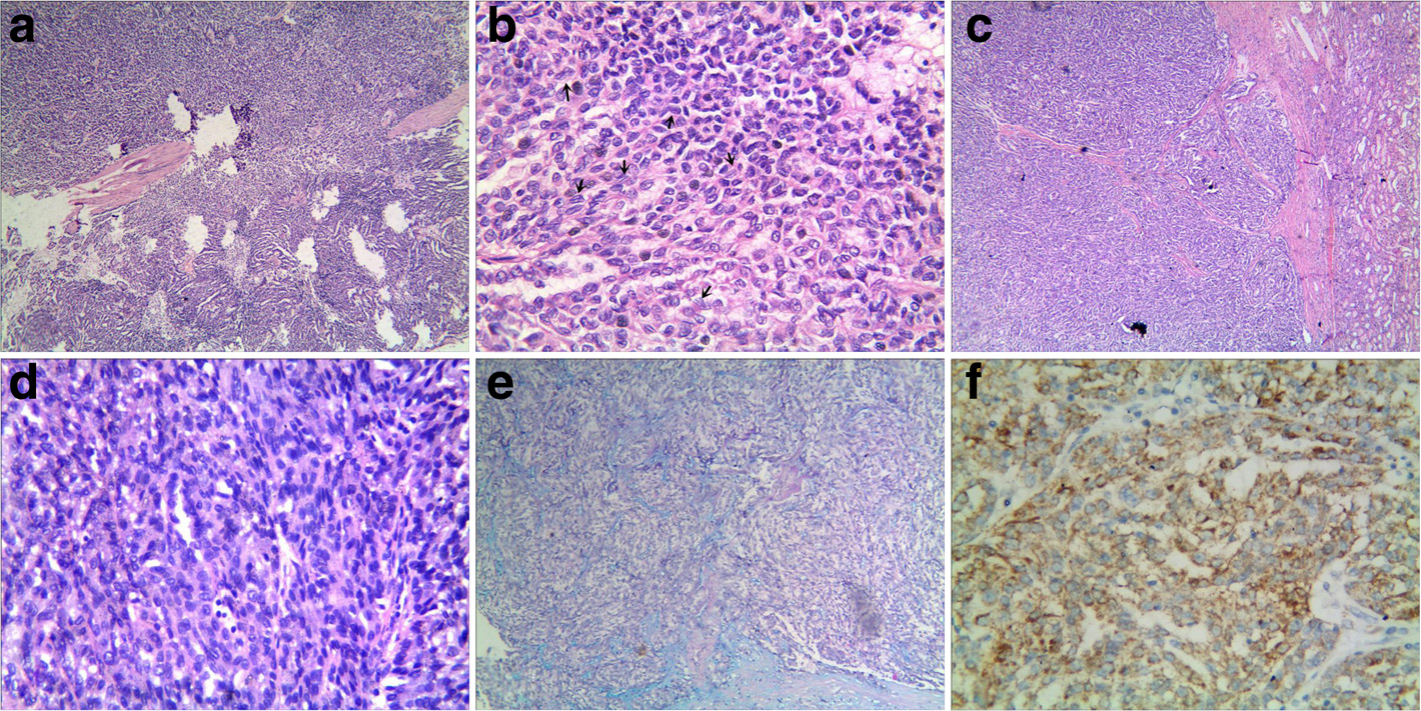
**Histological and immunohistochemical findings of sPRCC. (a)**: In cases of sPRCC, the tumor cells are arranged in a tubular pattern (×4). **(b)**: Nuclear grooves are observed in several cells (arrow) (×40). **(c)**: A fibrous septum is observed in cases of sPRCC (×4). **(d)**: The cells form solid sheets compressed against each other and have a spindle cell appearance (×40). **(e)**: Foci of mucin are found in case 3 (Alcian blue staining, ×10). **(f)**: Tumor cells were positive for AMACR in cases of both MTSC and sPRCC.

A fibrous septum was detected and predominantly tubular structure were seen in two cases of sPRCC. Tumor cells were cuboidal or oval with vacuolated chromatin and Fuhrman nuclear grade 2–3. Small nucleoli were observed in focal cells, with scarce light acidophilus cytoplasm. Nuclear grooves were also observed in several cells. Psammoma bodies and accumulation of foam cells were seen in the stroma. Papillary architecture was observed at the edge of the neoplasm, although they occupied <5% of the volume of the tumor. Focal tightly packed tubular structures of arranged in solid sheets showed similarities to spindle cells was seen in case 3. Alcian blue staining showed a focal area of mucin in case 3, although HE staining was inconspicuous (Figure 3).

Immunohistochemical findings showed that the tumor cells in all four cases were positive for AMACR, CK, CK7, CK19, EMA, HMWK, E-cadherin, and vimentin. Ki - 67 index was <5%. Other markers such as CD15, CD56, CgA, Syn and villin were negative in both. The two cases of MTSC were positive for NSE and negative for CD10 and RCC, whereas the two cases of sPRCC were negative for NSE, and only case 4 was CD10 and RCC positive. Alcian blue staining showed large amounts of mucin resembling mucinous stroma in two cases of MTSC and small amounts of mucin in the stroma of one case of sPRCC (case 3). The detailed results were listed in Table 3.

**Table 3:** **Immunohistochemical data of two cases of MTSC and sPRCC**

	**case**	**AMACR**	**CK**	**CK7**	**CK19**	**HMWK**	**EMA**	**E-ca**	**vim**	**Ki-67**	**CD10**	**AB**	**RCC**
MTSC	1	+	+	+	+	+	+	+	+	+,<5%	-	AB+	-
	2	+	+	+	+	+	+	+	+	+,<1%	-	AB+	-
sPRCC	3	+	+	+	+	+	+	+	+	+,<1%	-	AB+	-
	4	+	+	+	+	-	+	-	+	+,<5%	+	AB-	+
	case	NSE	villin	CD56	CgA	Syn	CD15						
MTSC	1	+	-	-	-	-	-						
	2	+	-	-	-	-	-						
sPRCC	3	-	-	-	-	-	-						
	4	-	-	-	-	-	-						

**Figure 4:**
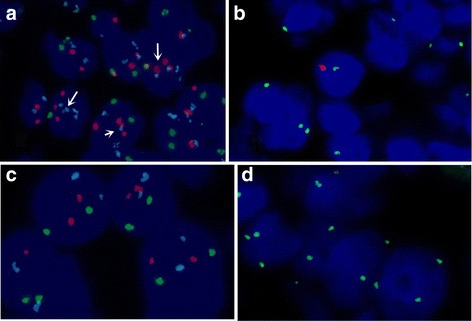
**Molecular genetics of two cases of MTSC. (a)**: FISH showed gains of chromosomes 7 (blue signal) and 17 (red signal) (arrow) in case 1. **(b)**: FISH showed loss of chromosome Y (red signal) in case 1. **(c)**: FISH showed normal chromosomes 7, 17. **(d)**: FISH showed normal X chromosome.

Gains of chromosomes 7, 17 and loss of Y which are characteristic of PRCC, were observed in two cases of sPRCC and one MTSC (case 1) (Figure 4). However, case 2 showed no gains of chromosomes 7, 17 and loss of Y. The detailed results were listed in Table 4.

**Table 4:** **Number and percentage of nuclei with fluorescent hybridization signals**

**Case**	**Chromosome**	**No. nuclei (% of total nuclei)**			
		**1 Signal(%)**	**2 Signal(%)**	**3 Signal(%)**	**4 Signal(%)**
1	7	13(21.66)	24(40.00)	17(28.33)	6(10.00)
	17	10(16.66)	31(51.66)	8(13.33)	11(18.33)
	Y	5(8.33)	0	0	0
2	7	17(28.33)	42(70.00)	1(1.666)	0
	17	19(31.66)	39(65.00)	2(3.33)	0
	Y	-	-	-	-
3	7	8(13.33)	35(58.33)	7(11.66)	7(11.66)
	17	9(15.00)	34(56.66)	13(21.66)	4(6.66)
	Y	9(15.00)	0	0	0
4	7	9(15.00)	26(43.33)	20(33.33)	5(8.33)
	17	6(10.00)	36(60.00)	8(13.33)	10(16.66)
	Y	7(11.66)	0	0	0

### Follow-up

During the follow-up examinations at 8 and 14 months, all four patients were alive with no evidence of recurrence or metastasis postoperatively. The patients continue to be followed up.

## Discussion

Our results proved that MTSC and sPRCC have overlapping morphological and immunohistochemical markers, including macroscopically, both of MTSC and sPRCC are well circumscribed, the cut surfaces are greyish-white in color. Histologically, these two kinds of tumors were both predominantly composed of tubular areas with a focal spindle cell appearance; tumor cells showed low nuclear grade; mucinous component, papillary architecture and foamy macrophages could be seen in both. Immunohistochemical assay demonstrated that the tumor cells of MTSC and sPRCC were positive for AMACR, CK, CK7, CK19, EMA, HMWK, E-cadherin, vimentin and Ki - 67 index was <5%. These results were similar to the previous studies [7-10,13]. Whereas some differences were observed such as psammoma bodies was only seen in sPRCC (case 3) and two cases of MTSC were negative for CD10 and RCC. However psammoma bodies could presented in classic MTSC and some cases of MTSC positive expression of CD10 and RCC were reported [5,7,8,10]. These findings suggested that MTSC and sPRCC have similar morphological and immunohistochemical features. Other differences were also observed such as the presence of a fibrous septum and nuclear grooves in sPRCC, which were not observed in MTSC. Two cases of MTSC were focally positive for NSE, which was consistent with the findings of some researchers reported that MTSC had neuroendocrine differentiation [14-16]. This change was not seen in sPRCC. However we did not find out that a fibrous septum, nuclear grooves and positivity for NSE could distinguish MTSC from sPRCC.

Molecular genetic analyses were performed to further evaluate these four cases. We found that case 1 (MTSC) and two cases of sPRCC both had gains of chromosomes 7 and 17 and loss of the Y chromosome. The results suggested that MTSC and sPRCC could have similar chromosomal abnormalities. A review of the literature indicated that MTSC was characterized by a variety of chromosomal abnormalities, including frequently losses of chromosomes 1, 4, 6, 8, 9, 11, 13, 14, 15, and 22 and gains of chromosomes 2, 5, 7, 9, 10, 11, 12, 16, 17, 18, 19, 20, 21, 22 and X [16-23]. Rakozy *et al*. [18] first demonstrated that MTSC showed losses of chromosomes 1, 4, 6, 8, 9, 13, 14, 15 and 22. Similar results had been reported subsequently. However Cossu-Rocca *et al.* [23] analyzed 10 cases of MTSC using interphase fluorescence in situ hybridization and showed lack of gains of chromosomes 7, 17 and loss of Y. In contrast, using comparative genomic hybridization (CGH), gains of chromosomes 7 and 17 were reported by others [16,17,19,21,22], but loss of the Y chromosome was never reported*.* The present study is the first reported case of MTSC which have loss of the Y chromosome. Compared with MTSC, a variety of chromosomal abnormalities in PRCC were reported, including losses of chromosomes 1, 4, 6, 9, 11, 13, 14, 18, Y and gains of chromosomes 7, 12, 16, 17, and 20 [24,25]. Jiang *et al*. [25] reported that typically, PRCC has gains of chromosomes 7, 16, 17 and frequently losses of chromosomes 1, 4, 6, 9, 13, 14, 18, X and Y. These findings provided evidence that MTSC has similar cytogenetic aberrations as those of PRCC showing. So we considered that genetically, MTSC was similar to PRCC.

MTSC is a low-grade malignant tumor, and only a few cases have been reported [26] that showed metastasis. sPRCC is similar, often presenting as a low-level renal tumor of low-clinical stage [27].

Our results and previously published data indicate that morphologic, immunophenotypic, molecular genetics and prognostic features show an overlap between MTSC with sPRCC. Therefore we conclude that MTSC is similar to sPRCC and may be a subtype of PRCC.

## Conclusion

MTSC and sPRCC have overlapping morphological and immunohistochemical markers, including a tubular structure, papillary architecture, spindle cell appearance, psammoma bodies, foamy macrophages, mucin and positive staining for vimentin, CK7, CK19, EMA, AMACR, CD10, and RCC. In addition, our results indicated that MTSC and sPRCC have similar chromosomal abnormalities. Therefore we conclude that MTSC is similar to sPRCC and maybe a subtype of PRCC. Because of the small number of cases reported, additional studies are necessary to confirm our conclusions.

## Patients’ consents

Written informed consents were obtained from the reviewed patients for publication of this case report and accompanying images. A copy of the written consent is available for review by the Editor-in Chief of this Journal.
